# Crystal structures of (*E*)-3-(4-hy­droxy­benzyl­idene)chroman-4-one and (*E*)-3-(3-hy­droxy­benzyl­idene)-2-phenyl­chroman-4-one

**DOI:** 10.1107/S2056989019015639

**Published:** 2019-11-22

**Authors:** Kamil Suchojad, Anna Dołęga, Angelika Adamus-Grabicka, Elżbieta Budzisz, Magdalena Małecka

**Affiliations:** a Department of Physical Chemistry, Faculty of Chemistry, University of Lodz, Pomorska 163/165, 90-236 Łódź, Poland; b Department of Inorganic Chemistry, Gdańsk University of Technology, G. Narutowicza 11/12., 80-233 Gdańsk, Poland; c Department of Cosmetic Raw Materials Chemistry, Faculty of Pharmacy, Medical University of Lodz, Muszynskiego 1, 90-151 Łódź, Poland

**Keywords:** crystal structure, chromanone derivative, flavanone derivative, lipophilicity index, Hirshfeld surface analysis

## Abstract

The synthesis and crystal structures of (*E*)-3-(4-hy­droxy­benzyl­idene)chroman-4-one, C_16_H_12_O_3_, **I**, and (*E*)-3-(3-hy­droxy­benzyl­idene)-2-phenyl­chroman-4-one, C_22_H_16_O_3_, **II**, are reported. These compounds are of inter­est with respect to biological activity. Both structures display inter­molecular C—H⋯O and O—H⋯O hydrogen bonding, forming layers in the crystal lattice. The crystal structure of compound **I** is consolidated by π–π inter­actions. The lipophilicity (log*P*) was determined as it is one of the parameters qualifying compounds as potential drugs. The log*P* value for compound **I** is associated with a larger contribution of C⋯H inter­action in the Hirshfeld surface.

## Abstract 2

Two biologically active compounds were synthesized and their crystal structures were determined. The characteristic feature of both structures is mol­ecular layers in the crystal lattice formed *via* C—H⋯O and O—H⋯O inter­actions. The mol­ecular Hirshfeld surfaces analysis were explored with two-dimensional fingerprint plots for the title compounds and other known structures from the literature. Additionally, the lipophilicity parameters (log*P*) were determined and related to the C⋯H contact contribution in the Hirshfeld surface.

## Chemical context   

Chromanone (chroman-4-one) and flavanone (2-phenyl­chroman-4-one) belong to the class of heterocyclic compounds and are composed of a benzene ring fused to a 2,3-di­hydro-γ-pyran­one ring (Emami & Ghanbarimasir, 2015 ▸). 3-Aryl­idenechromanones/flavanones and their derivatives are naturally occurring homoisoflavones, and can be obtained by condensing the corresponding aryl aldehydes with chromanone/flavanone. These compounds were synthesized for the first time by Robinson in the early 1920s by the condensation reaction of chromanone or flavanone with the appropriate aryl aldehyde using a catalyst (alcohol potassium hydroxide) (Perkin *et al.*,1926 ▸). In 1979, Levai and Schag synthesized *E*-3-aryl­idenechroman-4-one using piperidine as a catalyst (Levai & Schag, 1979 ▸). Several years later, in 1993, Pijewska and coworkers (Pijewska *et al.*, 1993 ▸) obtained the series of 3-aryl­ideneflavanones derivatives substituted by various groups using flavanones with aromatic aldehydes in the presence of piperidine. Flavonoid compounds belong to one of the largest and most inter­esting groups of chemical compounds. They are of inter­est to many scientists because they show biological properties (Nijveldt *et al.*, 2001 ▸; Williams *et al.*, 2004 ▸). Natural and synthetic flavonoids have a wide range of anti­oxidant, anti-allergic, anti-inflammatory, anti-microbial, anti-coagulant, anti-cholesterol or anti-cancer activities (Czaplińska *et al.*, 2012 ▸).
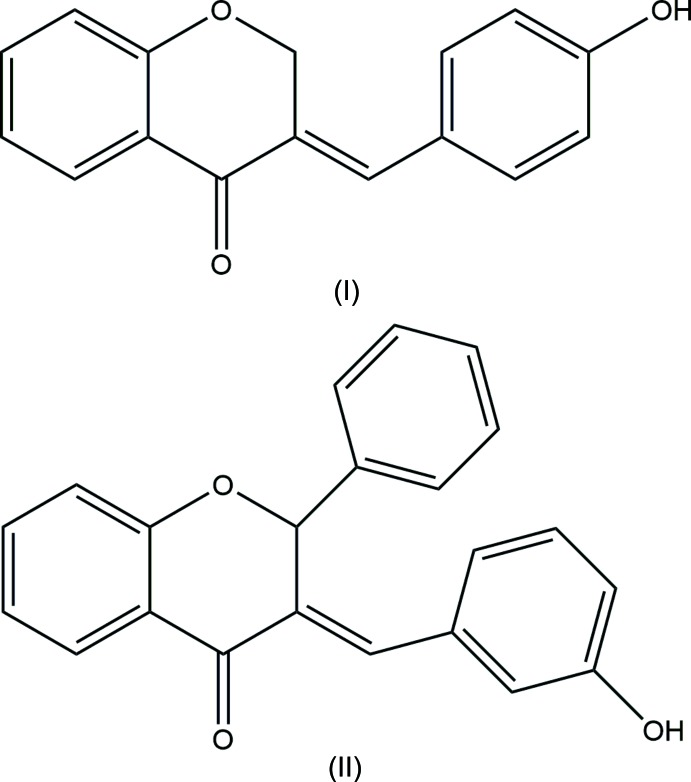


## Structural commentary   

**Figure 1:**
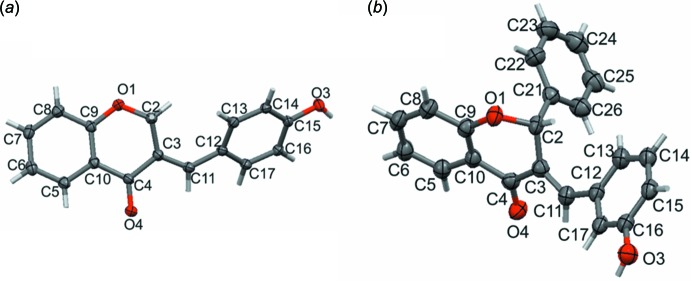
The mol­ecular structures of compounds **I** and **II** with displacement ellipsoids drawn at the 50% probability level.

**Figure 2:**
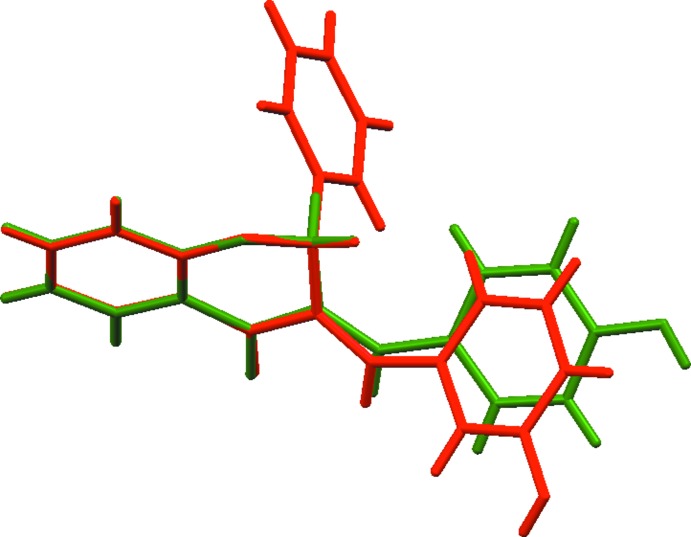
Overlay of compound **I** (green) and compound **II** (red).

The mol­ecular structures of **I** and **II** are shown in Fig. 1 ▸. The main chroman skeleton of each mol­ecule consists of a benzene ring fused with a pyran ring. In position 3 of the chroman moiety, a *para*-hy­droxy­benzyl­idene (**I**) or a *meta*-hy­droxy­benzyl­idene (**II**) substituent is connected to give the *E*-isomer, similar to the previously mentioned structure (Kupcewicz, *et al.*, 2013 ▸). Moreover in compound **II**, the chroman moiety is subsituted at position 2 by a phenyl ring. The pyran rings adopt an envelope conformation with puckering parameters *Q*_T_ = 0.371 (2) Å, φ_2_ = 233.8 (4)°, θ_2_ = 120.0 (3)° for **I**, and *Q*_T_ = 0.423 (3) Å, φ_2_ = 65.9 (5)°, θ_2_ = 58.5 (4)° for **II**. The dihedral angles between the hy­droxy­benzyl­idene rings and the main chroman skeleton are 47.54 (8) and 69.46 (12)°, respectively, for **I** and **II** (Fig. 2 ▸).

## Supra­molecular features   

**Figure 3:**
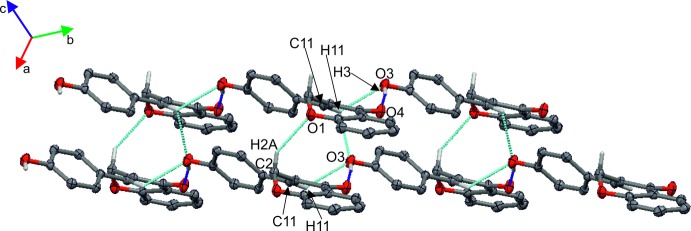
Partial packing of compound **I** showing the O—H⋯O (blue dotted lines) and C—H⋯O (cyan dotted lines) hydrogen-bonding network.

**Figure 4:**
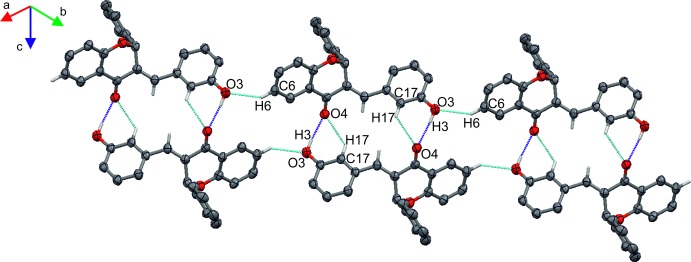
Partial packing of compound **II** showing the O—H⋯O (blue dotted lines) and C—H⋯O (cyan dotted lines) hydrogen-bonding network.

In the crystal packing of **I**, mol­ecules are connected into layers parallel to the *bc* plane *via* C—H⋯O and O—H⋯O hydrogen bonds (Table 1 ▸, Fig. 3 ▸). The stability of the layers is further enhanced by π–π stacking inter­actions occurring between the benzene rings fused with the pyran rings and the aromatic rings of adjacent hy­droxy­benzyl­idene groups (Table 2 ▸). In the crystal packing of **II**, mol­ecules are also linked by O—H⋯O and C—H⋯O hydrogen bonds (Table 3 ▸, Fig. 4 ▸) into layers parallel to the *ab* plane.

## Database survey   

**Figure 5:**
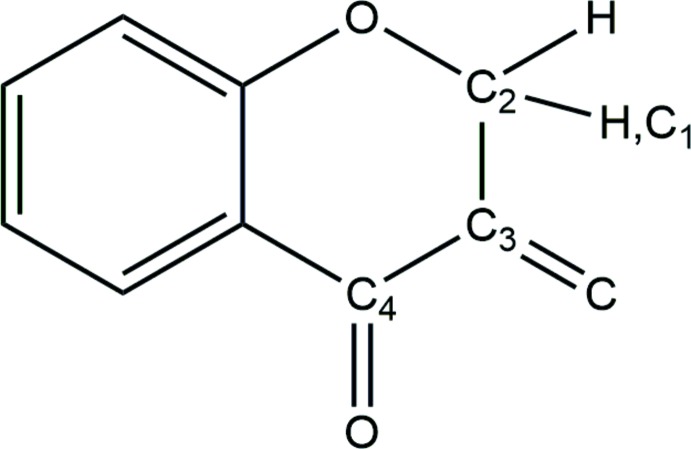
Reference moiety for database survey.

A search of the Cambridge Structural Database (CSD version 5.40, last update November 2018; Groom *et al.*, 2016 ▸) using the scheme presented in Fig. 5 ▸ found 41 chromanone (Ishikawa *et al.*, 2013*a* ▸,*b* ▸; Zimmerman *et al.*, 2015 ▸; Marx, Suresh *et al.*, 2007 ▸; Katrusiak *et al.*, 1987 ▸; Brien *et al.*, 2012 ▸; Suresh *et al.*, 2007 ▸; Boonsri *et al.*, 2005 ▸; Biruntha *et al.*, 2018 ▸; Talhi *et al.*, 2016 ▸; Wu, Liu *et al.*, 2011 ▸; Marx *et al.*, 2008 ▸; Cheng *et al.*, 2011 ▸; Valkonen *et al.*, 2012 ▸; Lepitre *et al.*, 2017 ▸; Gopaul, Shaikh, Koorbanally *et al.*, 2012 ▸; Gopaul, Koorbanally *et al.*, 2012 ▸; Marx, Manivannan *et al.*, 2007 ▸; Suresh *et al.*, 2007 ▸; Marx *et al.*, 2008 ▸; Hassaine *et al.*, 2016 ▸; Chantrapromma *et al.*, 2006 ▸; Zhang *et al.*, 2012 ▸; Augustine *et al.*, 2008 ▸; Gopaul, Shaikh, Ramjugernath *et al.*, 2012 ▸; Gopaul & Koorbanally, 2012 ▸; Zhang *et al.*, 2013 ▸) and four flavanone structures (Zhong *et al.*, 2013 ▸; Kupcewicz *et al.*, 2013 ▸; Wu, Zeng *et al.*, 2011 ▸; Monserrat *et al.*, 2013 ▸). In the flavanone structures, the phenyl substituent at the C*2* position is always nearly perpendicular to the chroman moiety, with the C(phen)—C2—C3—C4 torsion angle in the range 82.44–107.90°. In both chromanone and flavanone structures, the pyran ring adopts a slightly distorted envelope conformation. In the 41 chromanone derivatives, the bond distances and angles within the chroman moiety are in good agreement with those found in compound **I**.

## Experimental and theoretical lipophilicity of compounds I and II   

Lipophilicity is one of the descriptors that is currently used in the design of new drugs and in assessing the activity of medicinal substances (Jóźwiak *et al.*, 2001 ▸). Most often, the increase in lipophilicity increases the biological activity of compounds as a result of the affinity of substances with biological membranes and better permeability (Dołowy, 2009 ▸). However, a further increase in lipophilicity results in greater affinity for lipids and hinders the transport of compound mol­ecules through the aqueous phase. That is why it is important to choose substances with optimal hydro­phobic and hydro­philic properties and partition coefficient log*P* (Dołowy, 2009 ▸).

The experimental lipophilicity (log*P*) of compounds **I** and **II** was determined using the RP–TLC method. The values of log*P* obtained are 2.95 and 3.98, respectively for **I** and **II**, the difference being due to the different, bulky substituent at the C2 position of the pyran ring. The theoretical values of lipophilicity (miLog*P)* also show the same trend, the value for compound **I** is lower (miLog*P =* 3.14) than that for compound **II** (miLog*P* = 4.70). This is in agreement with the values previously reported for similar aryl­idenochromanone/flavanone derivatives (Adamus-Grabicka *et al.*, 2018 ▸). The theoretical values of lipophilicity were calculated using the online *Molinspiration Cheminformatics* software (http://www.molinspiration.com). According to the ‘rule of five’ proposed by Lipinski *et al.* (2001 ▸), compounds **I** and **II** may be potential anti-cancer drugs, the most important parameters according to Lipinski being the log*P* value (logP < 5) and molar mass (< 500 Da).

## Hirshfeld surface analysis and lipophilicity index *versus* C⋯H contact   

As the Hirshfeld surface (HS) analysis may provide useful descriptors for QSAR study (Kupcewicz, *et al.*, 2016 ▸) and the lipophilicity parameter in biologically active compounds is associated with the contribution of inter­molecular inter­actions to the Hirshfeld surface (Małecka & Budzisz, 2014 ▸), we generated the Hirshfeld surfaces (Hirshfeld, 1977 ▸; Spackman & Jayatilaka, 2009 ▸) using the *CrystalExplorer* program (Turner *et al.*, 2017 ▸) for chromone and flavanone derivatives for which the lipophilicity parameters are available, *i.e*. compound **I**, **II**, 3-(4-chloro­benzyl­idene)-2-phenyl-2,3-di­hydro-4*H*-chromen-4-one (**III**; Kupcewicz *et al.*, 2013 ▸), (*E*)-3-(4-*N,N*-di­ethyl­amino­benzyl­idene)chroman-4-one (**IV**; Adam­us-Grabicka *et al.*, 2018 ▸) and (*E*)-3-(4-*N*,*N*-di­ethyl­amino­benzyl­idene)-2-phenyl­chroman-4-one (**V**; Adamus-Grabicka *et al.*, 2018 ▸).

**Figure 6:**
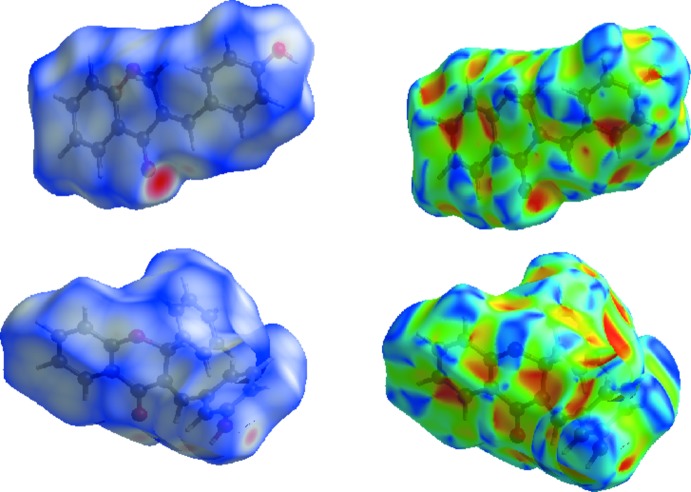
View of the three-dimensional Hirshfeld surfaces of the title compounds plotted over *d*_norm_ (left) and shape-index (right); first row: compound **I**, second row: compound **II**.

**Figure 7:**
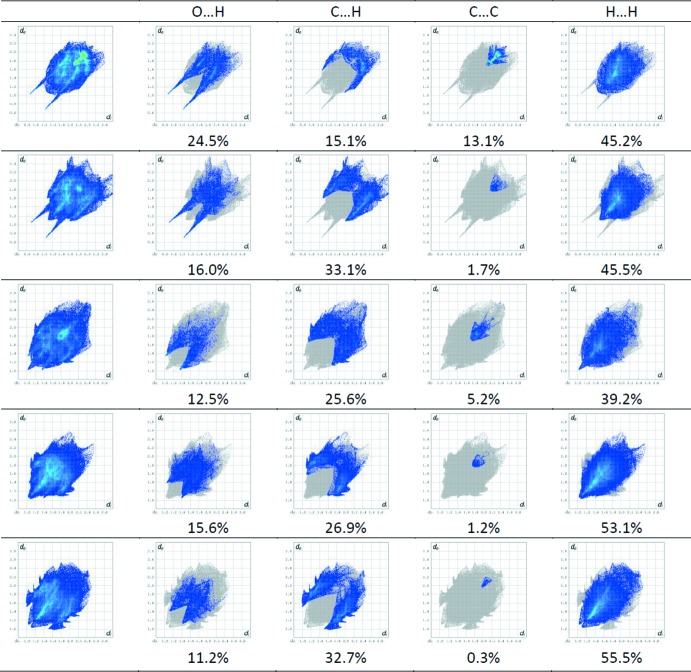
Fingerprint plots of the title compounds; full Hirshfeld surface (left) and delineated into H⋯O, H⋯C, C⋯C, and H⋯H contacts, showing the percentage contributions of the contacts to the total Hirshfeld surface area of the mol­ecules. First row: compound **I**; second row: compound **II**; third row: 3-(4-chloro­benzyl­idene)-2-phenyl-2,3-di­hydro-4*H*-chromen-4-one (Kupcewicz *et al.*, 2013 ▸); fourth row: (*E*)-3-(4-*N,N*-di­ethyl­amino­benzyl­idene)chroman-4-one (Adamus-Grabicka *et al.*, 2018 ▸); fifth row: (*E*)-3-(4-*N*,*N*-di­ethyl­amino­benzyl­idene)-2-phenyl­chroman-4-one (Adamus-Grabicka *et al.*, 2018 ▸).

The Hirshfeld surfaces were mapped over *d*_norm_ (Fig. 6 ▸). The red, white and blue regions visible on the *d*_norm_ surfaces indicate contacts with distances shorter, longer and equal to the van der Waals radii. The decomposition of the HS into 2D fingerprint plots for particular contacts is presented in Fig. 7 ▸, together with the relative percentage of contributions of different contacts. The dominant inter­action in all derivatives is the H⋯H inter­action. The contribution to the Hirshfeld surface is in the range 39.2– 55.5% for **III** and **V**. Comparing the C⋯C contacts, we can observe a large spread of percentage contribution ranging from 0.3% for **V** to 13.1% for compound **I**. This is also reflected in the presence of π–π stacking inter­actions observed in compound **I** (Table 2 ▸).

As in our previous studies (Małecka *et al.*, 2014 ▸; Kupcewicz *et al.*, 2103), we found a relationship between the log*P* value and the fraction of the Hirshfeld surface covered by different inter­molecular inter­actions. The increase of log*P* corresponds in fact to increasing the C⋯H contribution in the Hirshfeld surface. Furthermore, for compounds **I**–**V**, the contribution of the O⋯H inter­action in the Hirshfeld surface is inversely proportional to the value of log*P*.

## Synthesis and crystallization   

**Figure 8:**
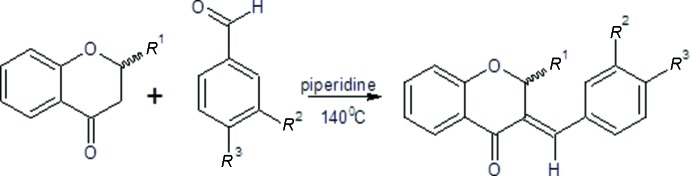
Scheme of the synthesis of compounds **I** and **II**. *R* ^1^ = H/Ph, *R* ^2^ = H/OH, *R* ^3^ = OH/H, respectively for compound **I** and **II**.

The synthesis of compounds **I** and **II** is based on the condensation of chromanone or flavanone with an aryl aldehyde in the presence of piperidine (Fig. 8 ▸). Compound **I** was prepared according to a slightly modified procedure with respect to that described in the literature (Levai & Schag, 1979 ▸). A mechanically stirred mixture of chroman-4-one (0.01 mol), *p*-meth­oxy­benzaldehyde (0.01 mol) and five drops of piperidine was heated at 413 K in an oil bath for four h. The progress of the synthesis was controlled by thin layer chromatography (TLC) using toluene/methanol (9:1 *v*/*v*) as eluent. After cooling the reaction mixture was left for 24 h at room temperature. The solidified product was filtered and crystallized from methanol. Compound **I** was obtained as a yellow powder. The isolated solid was further recrystallized by slow evaporation at room temperature of an acetone solution. Yield: 64%, M.p.: 501–502.5 K. MS (ESI^+^): *m*/*z* 253.3 C_16_H_12_O_3_ [*M*+H]^+^. IR (KBr): 3126 (O—H), 2809 (C—H_aromat_), 1652 (C=O), 1608, 1578 (C=C), 1164 (C–O—C), 751 (=C—H). ^1^ *H* NMR (600 MHz, DMSO-*d*_6_) δ (ppm): 5.42 (1H, *s*, =CH), 6.86–7.86 (8H, *m*, C—H _aromat_), 7.87 (2H, *d*, *J*_AB_ = 18 Hz C2—H), 10.12 (1H, *s*, OH). Analysis calculated for C_16_H_12_O_3_ (*M* = 252.23 g mol^−1^) % C: 76.18; % H: 4.81; % O: 19.01. Found % C: 75.3; % H: 5.01; % O: 19.69.

Compound **II** was synthesized according to the procedure described by Pijewska *et al.*, (1993 ▸). A mixture of 2-phenyl­chroman-4-one (0.01 mol), 3-hy­droxy­benzaldehyde (0.01 mol) and five drops of piperidine was heated under reflux in an oil bath with mechanical stirring. The reaction proceeded at 413 K for 5 h. The progress of the reaction was controlled by TLC (eluent: toluene/methanol, 9:1 *v*/*v*). After cooling at room temperature, the mixture was dissolved in methanol. After 24 h compound **II** precipitated as a light-cream fine crystalline powder and was purified by crystallization from methanol. Crystal suitable for X-ray analysis were obtained by slow evaporation of an ethanol solution at room temperature. Yield: 52.4%. M.p.: 482–483 K. MS (ESI^+^): *m*/*z* 329.2 C_22_H_16_O_3_ [M+H]^+^. IR (KBr): 3297 (O—H), 3054 (C—H_aromat_), 2351 (C—H_aliph_), 1663 (C=O), 1608, 1590, 1504 (C=C), 1141 (C—O—C), 757 (=C—H). ^1^ *H* NMR (600 MHz, DMSO-*d*_6_) δ (ppm): 6.57 (1H, *s*, C2—H), 5.69 (1H, *s*, =CH), 6.89–7.91 (14H, *m*, CH_aromat_), 8.12 (1H, *s*, OH). Analysis calculated For C_22_H_16_O_3_ (*M* = 328.19 g mol^−1^) %C: 80.51; %H: 4.87; % O: 14.62. Found %C: 79.99; %H: 5.11; % O: 14.90.

## Refinement   

Crystal data, data collection and structure refinement details are summarized in Table 4 ▸. All hydrogen atoms were fixed geometrically at calculated positions (O—H = 0.84 Å, C—H = 0.95–0.99 Å) and refined as riding model with *U*_iso_(H) = 1.5*U*_eq_(O) or 1.2*U*_eq_(C). A rotating model was used for the hy­droxy groups.

## Supplementary Material

Crystal structure: contains datablock(s) I, II. DOI: 10.1107/S2056989019015639/rz5266sup1.cif

Structure factors: contains datablock(s) I. DOI: 10.1107/S2056989019015639/rz5266Isup4.hkl

Structure factors: contains datablock(s) II. DOI: 10.1107/S2056989019015639/rz5266IIsup5.hkl

Supporting information file. DOI: 10.1107/S2056989019015639/rz5266Isup4.cml

Supporting information file. DOI: 10.1107/S2056989019015639/rz5266IIsup5.cml

CCDC references: 1966749, 1966750

Additional supporting information:  crystallographic information; 3D view; checkCIF report

## Figures and Tables

**Table 1:** Hydrogen-bond geometry (Å, °) for **I** [Chem scheme1]

*D*—H⋯*A*	*D*—H	H⋯*A*	*D*⋯*A*	*D*—H⋯*A*
C11—H11⋯O3^i^	0.95	2.55	3.264 (2)	132
C11—H11⋯O3^ii^	0.95	2.52	3.194 (2)	129
O3—H3⋯O4^iii^	0.84	1.85	2.6852 (19)	172
C2—H2*A*⋯O1^iv^	0.99	2.53	3.397 (3)	147
C11—H11⋯O4	0.95	2.45	2.818 (2)	103

Symmetry codes: (i) 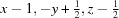; (ii) 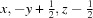; (iii) 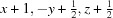; (iv) 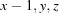.

**Table 2:** Geometrical parameters (Å, °) for the π–π stacking inter­actions for compound **I** *Cg*(1) and *Cg*(2) are the centroids of the C5–C10 and C12–C17 rings, respectively; α refers to the dihedral angle between planes (I) and (J); β refers to the angle between the *Cg*(*I*))–*Cg*(*J*) vector and normal to plane (*I*); γ refers to the angle between the *Cg*(*I*))–*Cg*(*J*) vector and normal to plane (*J*).

	*Cg*(*I*)⋯*Cg*(*J*)	*Cg*(*I*)_Perp	*Cg*(*J*)_Perp	α	β	γ
*Cg*(1)⋯*Cg*(1)^i^	3.8508 (13)	3.5260 (9)	−3.5259 (9)	0.03 (10)	23.7	23.7
*Cg*(1)⋯*Cg*(1)^ii^	3.8512 (13)	3.5260 (9)	−3.5262 (9)	0.03 (10)	23.7	23.7
*Cg*(2)⋯*Cg*(2)^i^	3.8510 (13)	3.3739 (8)	−3.3738 (8)	0.03 (10)	28.8	28.8
*Cg*(2)⋯*Cg*(2)^ii^	3.8510 (13)	3.3740 (8)	−3.3738 (8)	0.03 (10)	28.8	28.8

Symmetry codes: (i) −1 + *x*, *y*, *z*; (ii) 1 + *x*, *y*, *z*.

**Table 3:** Hydrogen-bond geometry (Å, °) for **II** [Chem scheme1]

*D*—H⋯*A*	*D*—H	H⋯*A*	*D*⋯*A*	*D*—H⋯*A*
O3—H3⋯O4^i^	0.84	1.89	2.728 (3)	172
C17—H17⋯O4^i^	0.95	2.49	3.184 (4)	130
C6—H6⋯O3^ii^	0.95	2.45	3.265 (4)	143
C11—H11⋯O4	0.95	2.43	2.807 (3)	103

Symmetry codes: (i) 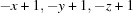; (ii) 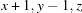.

**Table 4:** Experimental details

	**I**	**II**
Crystal data		
Chemical formula	C_16_H_12_O_3_	C_22_H_16_O_3_
*M*_r_	252.27	328.37
Crystal system, space group	Monoclinic, *P*2_1_/*c*	Triclinic, *P* 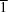
Temperature (K)	120	120
*a*, *b*, *c* (Å)	3.8510 (2), 22.2541 (11), 13.7837 (9)	5.3969 (6), 11.6576 (16), 12.944 (2)
α, β, γ (°)	90, 96.766 (5), 90	91.992 (12), 98.282 (10), 97.568 (10)
*V* (Å^3^)	1173.04 (11)	797.68 (19)
*Z*	4	2
Radiation type	Mo *K*α	Mo *K*α
μ (mm^−1^)	0.10	0.09
Crystal size (mm)	0.4 × 0.2 × 0.1	0.8 × 0.2 × 0.05

Data collection		
Diffractometer	STOE IPDS 2T	STOE IPDS 2T
Absorption correction	–	–
No. of measured, independent and observed [*I* > 2σ(*I*)] reflections	7027, 2413, 1618	6849, 3281, 1804
*R*_int_	0.050	0.077
(sin θ/λ)_max_ (Å^−1^)	0.628	0.628

Refinement		
*R*[*F* ^2^ > 2σ(*F* ^2^)], *wR*(*F* ^2^), *S*	0.048, 0.115, 1.03	0.068, 0.200, 0.94
No. of reflections	2413	3281
No. of parameters	173	228
H-atom treatment	H-atom parameters constrained	H-atom parameters constrained
Δρ_max_, Δρ_min_ (e Å^−3^)	0.19, −0.19	0.23, −0.29

Computer programs: *X-AREA* and *X-RED32* (Stoe & Cie, 2002 ▸), *SHELXT* (Sheldrick, 2015*a* ▸), *SHELXL2014/7* (Sheldrick, 2015*b* ▸) and *publCIF* (Westrip, 2010 ▸).

## supplementary crystallographic information

### (E)-3-(4-Hydroxybenzylidene)chroman-4-one (I) Crystal data

**Table:**

C_16_H_12_O_3_	*D*_x_ = 1.428 Mg m^−^^3^
*M**_r_* = 252.27	Melting point: 220 K
Monoclinic, *P*2_1_/*c*	Mo *K*α radiation, λ = 0.71073 Å
*a* = 3.8510 (2) Å	Cell parameters from 219 reflections
*b* = 22.2541 (11) Å	θ = 4.1–28.9°
*c* = 13.7837 (9) Å	µ = 0.10 mm^−^^1^
β = 96.766 (5)°	*T* = 120 K
*V* = 1173.04 (11) Å^3^	Needle, light-yellow
*Z* = 4	0.4 × 0.2 × 0.1 mm
*F*(000) = 528	

### (E)-3-(4-Hydroxybenzylidene)chroman-4-one (I) Data collection

**Table:**

STOE IPDS 2T diffractometer	1618 reflections with *I* > 2σ(*I*)
Radiation source: GeniX Mo, 0.05 x 0.05 mm2 microfocus	*R*_int_ = 0.050
Detector resolution: 6.67 pixels mm^-1^	θ_max_ = 26.5°, θ_min_ = 3.7°
rotation method, ω scans	*h* = −4→4
7027 measured reflections	*k* = −27→27
2413 independent reflections	*l* = −16→17

### (E)-3-(4-Hydroxybenzylidene)chroman-4-one (I) Refinement

**Table:**

Refinement on *F*^2^	Primary atom site location: difference Fourier map
Least-squares matrix: full	Secondary atom site location: difference Fourier map
*R*[*F*^2^ > 2σ(*F*^2^)] = 0.048	Hydrogen site location: inferred from neighbouring sites
*wR*(*F*^2^) = 0.115	H-atom parameters constrained
*S* = 1.02	*w* = 1/[σ^2^(*F*_o_^2^) + (0.0546*P*)^2^ + 0.2287*P*] where *P* = (*F*_o_^2^ + 2*F*_c_^2^)/3
2413 reflections	(Δ/σ)_max_ < 0.001
173 parameters	Δρ_max_ = 0.19 e Å^−^^3^
0 restraints	Δρ_min_ = −0.19 e Å^−^^3^

### (E)-3-(4-Hydroxybenzylidene)chroman-4-one (I) Special details

**Table:**

Geometry. All esds (except the esd in the dihedral angle between two l.s. planes) are estimated using the full covariance matrix. The cell esds are taken into account individually in the estimation of esds in distances, angles and torsion angles; correlations between esds in cell parameters are only used when they are defined by crystal symmetry. An approximate (isotropic) treatment of cell esds is used for estimating esds involving l.s. planes.

### (E)-3-(4-Hydroxybenzylidene)chroman-4-one (I) Fractional atomic coordinates and isotropic or equivalent isotropic displacement parameters (Å^2^)

**Table:**

	*x*	*y*	*z*	*U*_iso_*/*U*_eq_	
O3	0.5127 (4)	0.21588 (6)	0.68339 (10)	0.0282 (4)	
H3	0.5952	0.1823	0.6701	0.042*	
O4	−0.1988 (4)	0.39218 (6)	0.15977 (10)	0.0304 (4)	
O1	0.2599 (4)	0.50810 (6)	0.36926 (10)	0.0252 (4)	
C9	0.1347 (5)	0.52849 (8)	0.27851 (14)	0.0217 (4)	
C17	0.3626 (5)	0.25884 (8)	0.42639 (15)	0.0218 (4)	
H17	0.4012	0.2441	0.3639	0.026*	
C3	0.0393 (5)	0.40641 (8)	0.32620 (14)	0.0207 (4)	
C14	0.2335 (5)	0.29994 (8)	0.60754 (15)	0.0216 (4)	
H14	0.1846	0.3135	0.6698	0.026*	
C11	0.0949 (5)	0.34735 (8)	0.34238 (15)	0.0219 (4)	
H11	0.0614	0.3229	0.2856	0.026*	
C10	−0.0381 (5)	0.49118 (8)	0.20685 (15)	0.0217 (4)	
C12	0.1982 (5)	0.31486 (8)	0.43307 (14)	0.0200 (4)	
C13	0.1298 (5)	0.33398 (8)	0.52539 (15)	0.0219 (4)	
H13	0.0104	0.3709	0.5318	0.026*	
C8	0.2011 (6)	0.58826 (9)	0.25768 (15)	0.0256 (5)	
H8	0.3229	0.6133	0.3061	0.031*	
C15	0.4089 (5)	0.24590 (8)	0.59954 (15)	0.0208 (4)	
C7	0.0889 (6)	0.61086 (9)	0.16621 (16)	0.0277 (5)	
H7	0.1330	0.6518	0.1523	0.033*	
C4	−0.0782 (5)	0.42671 (9)	0.22612 (14)	0.0216 (4)	
C2	0.0920 (6)	0.45481 (8)	0.40230 (15)	0.0227 (4)	
H2A	−0.1381	0.4662	0.4220	0.027*	
H2B	0.2363	0.4387	0.4607	0.027*	
C6	−0.0882 (6)	0.57486 (9)	0.09380 (16)	0.0283 (5)	
H6	−0.1649	0.5909	0.0311	0.034*	
C5	−0.1499 (6)	0.51545 (9)	0.11493 (15)	0.0249 (5)	
H5	−0.2708	0.4906	0.0661	0.030*	
C16	0.4693 (5)	0.22483 (8)	0.50797 (15)	0.0217 (4)	
H16	0.5830	0.1874	0.5018	0.026*	

### (E)-3-(4-Hydroxybenzylidene)chroman-4-one (I) Atomic displacement parameters (Å^2^)

**Table:**

	*U*^11^	*U*^22^	*U*^33^	*U*^12^	*U*^13^	*U*^23^
O3	0.0407 (9)	0.0236 (7)	0.0200 (8)	0.0065 (7)	0.0022 (7)	0.0031 (6)
O4	0.0459 (10)	0.0235 (7)	0.0198 (8)	−0.0049 (7)	−0.0044 (7)	−0.0004 (6)
O1	0.0305 (8)	0.0209 (7)	0.0231 (8)	−0.0046 (6)	−0.0020 (6)	0.0006 (6)
C9	0.0221 (11)	0.0231 (10)	0.0200 (11)	0.0031 (8)	0.0023 (8)	0.0014 (8)
C17	0.0266 (11)	0.0229 (10)	0.0160 (10)	−0.0013 (8)	0.0025 (8)	−0.0018 (8)
C3	0.0204 (10)	0.0223 (9)	0.0190 (11)	−0.0003 (8)	0.0015 (8)	0.0008 (8)
C14	0.0257 (11)	0.0199 (9)	0.0192 (11)	−0.0036 (8)	0.0030 (8)	−0.0029 (7)
C11	0.0229 (11)	0.0242 (10)	0.0179 (11)	−0.0002 (8)	−0.0003 (8)	−0.0023 (8)
C10	0.0240 (11)	0.0200 (9)	0.0212 (11)	0.0025 (8)	0.0036 (9)	0.0006 (8)
C12	0.0205 (10)	0.0215 (9)	0.0178 (10)	−0.0019 (8)	0.0009 (8)	−0.0008 (8)
C13	0.0218 (10)	0.0192 (10)	0.0245 (11)	−0.0005 (8)	0.0015 (8)	−0.0001 (8)
C8	0.0271 (12)	0.0228 (10)	0.0276 (12)	−0.0012 (8)	0.0052 (9)	−0.0029 (8)
C15	0.0228 (10)	0.0198 (9)	0.0190 (10)	−0.0023 (8)	−0.0010 (8)	0.0035 (8)
C7	0.0345 (13)	0.0200 (10)	0.0303 (13)	0.0021 (9)	0.0111 (10)	0.0028 (8)
C4	0.0235 (11)	0.0234 (10)	0.0169 (11)	0.0013 (8)	−0.0014 (8)	−0.0001 (8)
C2	0.0280 (11)	0.0203 (9)	0.0193 (11)	−0.0016 (8)	0.0000 (9)	0.0005 (8)
C6	0.0326 (12)	0.0275 (11)	0.0256 (12)	0.0066 (9)	0.0070 (9)	0.0055 (9)
C5	0.0285 (12)	0.0250 (10)	0.0211 (11)	0.0024 (8)	0.0024 (9)	−0.0008 (8)
C16	0.0249 (11)	0.0169 (9)	0.0231 (11)	0.0006 (8)	0.0024 (9)	−0.0005 (8)

### (E)-3-(4-Hydroxybenzylidene)chroman-4-one (I) Geometric parameters (Å, º)

**Table:**

O3—C15	1.354 (2)	C11—H11	0.9500
O3—H3	0.8400	C10—C5	1.398 (3)
O4—C4	1.242 (2)	C10—C4	1.471 (3)
O1—C9	1.364 (2)	C12—C13	1.396 (3)
O1—C2	1.450 (2)	C13—H13	0.9500
C9—C8	1.391 (3)	C8—C7	1.379 (3)
C9—C10	1.397 (3)	C8—H8	0.9500
C17—C16	1.378 (3)	C15—C16	1.392 (3)
C17—C12	1.406 (3)	C7—C6	1.394 (3)
C17—H17	0.9500	C7—H7	0.9500
C3—C11	1.346 (3)	C2—H2A	0.9900
C3—C4	1.471 (3)	C2—H2B	0.9900
C3—C2	1.501 (3)	C6—C5	1.381 (3)
C14—C13	1.382 (3)	C6—H6	0.9500
C14—C15	1.390 (3)	C5—H5	0.9500
C14—H14	0.9500	C16—H16	0.9500
C11—C12	1.458 (3)		
			
C15—O3—H3	109.5	C7—C8—H8	120.3
C9—O1—C2	115.95 (15)	C9—C8—H8	120.3
O1—C9—C8	116.97 (18)	O3—C15—C14	117.16 (18)
O1—C9—C10	122.55 (17)	O3—C15—C16	122.95 (18)
C8—C9—C10	120.40 (18)	C14—C15—C16	119.89 (18)
C16—C17—C12	121.80 (19)	C8—C7—C6	121.30 (19)
C16—C17—H17	119.1	C8—C7—H7	119.4
C12—C17—H17	119.1	C6—C7—H7	119.4
C11—C3—C4	118.72 (18)	O4—C4—C10	120.57 (18)
C11—C3—C2	125.41 (18)	O4—C4—C3	123.19 (18)
C4—C3—C2	115.86 (16)	C10—C4—C3	116.22 (17)
C13—C14—C15	120.37 (19)	O1—C2—C3	113.35 (16)
C13—C14—H14	119.8	O1—C2—H2A	108.9
C15—C14—H14	119.8	C3—C2—H2A	108.9
C3—C11—C12	130.40 (19)	O1—C2—H2B	108.9
C3—C11—H11	114.8	C3—C2—H2B	108.9
C12—C11—H11	114.8	H2A—C2—H2B	107.7
C5—C10—C9	118.78 (18)	C5—C6—C7	118.8 (2)
C5—C10—C4	120.86 (18)	C5—C6—H6	120.6
C9—C10—C4	120.22 (18)	C7—C6—H6	120.6
C13—C12—C17	117.70 (18)	C6—C5—C10	121.3 (2)
C13—C12—C11	124.65 (18)	C6—C5—H5	119.4
C17—C12—C11	117.57 (17)	C10—C5—H5	119.4
C14—C13—C12	120.83 (18)	C17—C16—C15	119.31 (18)
C14—C13—H13	119.6	C17—C16—H16	120.3
C12—C13—H13	119.6	C15—C16—H16	120.3
C7—C8—C9	119.48 (19)		

### (E)-3-(4-Hydroxybenzylidene)chroman-4-one (I) Hydrogen-bond geometry (Å, º)

**Table:**

*D*—H···*A*	*D*—H	H···*A*	*D*···*A*	*D*—H···*A*
C11—H11···O3^i^	0.95	2.55	3.264 (2)	132
C11—H11···O3^ii^	0.95	2.52	3.194 (2)	129
O3—H3···O4^iii^	0.84	1.85	2.6852 (19)	172
C2—H2*A*···O1^iv^	0.99	2.53	3.397 (3)	147
C11—H11···O4	0.95	2.45	2.818 (2)	103

Symmetry codes: (i) *x*−1, −*y*+1/2, *z*−1/2; (ii) *x*, −*y*+1/2, *z*−1/2; (iii) *x*+1, −*y*+1/2, *z*+1/2; (iv) *x*−1, *y*, *z*.

### (E)-3-(3-Hydroxybenzylidene)-2-phenylchroman-4-one (II) Crystal data

**Table:**

C_22_H_16_O_3_	*F*(000) = 344
*M**_r_* = 328.37	*D*_x_ = 1.367 Mg m^−^^3^
Triclinic, *P*1	Melting point: 210 K
*a* = 5.3969 (6) Å	Mo *K*α radiation, λ = 0.71073 Å
*b* = 11.6576 (16) Å	Cell parameters from 3650 reflections
*c* = 12.944 (2) Å	θ = 3.5–29.5°
α = 91.992 (12)°	µ = 0.09 mm^−^^1^
β = 98.282 (10)°	*T* = 120 K
γ = 97.568 (10)°	Plate, colourless
*V* = 797.68 (19) Å^3^	0.8 × 0.2 × 0.05 mm
*Z* = 2	

### (E)-3-(3-Hydroxybenzylidene)-2-phenylchroman-4-one (II) Data collection

**Table:**

STOE IPDS 2T diffractometer	1804 reflections with *I* > 2σ(*I*)
Radiation source: GeniX Mo, 0.05 x 0.05 mm2 microfocus	*R*_int_ = 0.077
Detector resolution: 6.67 pixels mm^-1^	θ_max_ = 26.5°, θ_min_ = 3.5°
rotation method, ω scans	*h* = −6→6
6849 measured reflections	*k* = −13→14
3281 independent reflections	*l* = −16→16

### (E)-3-(3-Hydroxybenzylidene)-2-phenylchroman-4-one (II) Refinement

**Table:**

Refinement on *F*^2^	Primary atom site location: difference Fourier map
Least-squares matrix: full	Secondary atom site location: difference Fourier map
*R*[*F*^2^ > 2σ(*F*^2^)] = 0.068	Hydrogen site location: inferred from neighbouring sites
*wR*(*F*^2^) = 0.200	H-atom parameters constrained
*S* = 0.94	*w* = 1/[σ^2^(*F*_o_^2^) + (0.1159*P*)^2^] where *P* = (*F*_o_^2^ + 2*F*_c_^2^)/3
3281 reflections	(Δ/σ)_max_ < 0.001
228 parameters	Δρ_max_ = 0.23 e Å^−^^3^
0 restraints	Δρ_min_ = −0.29 e Å^−^^3^

### (E)-3-(3-Hydroxybenzylidene)-2-phenylchroman-4-one (II) Special details

**Table:**

Geometry. All esds (except the esd in the dihedral angle between two l.s. planes) are estimated using the full covariance matrix. The cell esds are taken into account individually in the estimation of esds in distances, angles and torsion angles; correlations between esds in cell parameters are only used when they are defined by crystal symmetry. An approximate (isotropic) treatment of cell esds is used for estimating esds involving l.s. planes.

### (E)-3-(3-Hydroxybenzylidene)-2-phenylchroman-4-one (II) Fractional atomic coordinates and isotropic or equivalent isotropic displacement parameters (Å^2^)

**Table:**

	*x*	*y*	*z*	*U*_iso_*/*U*_eq_	
O1	0.1107 (4)	0.13471 (17)	0.24184 (16)	0.0423 (6)	
O4	0.7889 (4)	0.30522 (17)	0.39197 (16)	0.0432 (6)	
O3	−0.1593 (4)	0.6939 (2)	0.44060 (17)	0.0503 (6)	
H3	−0.0545	0.6964	0.4956	0.075*	
C10	0.5289 (5)	0.1266 (2)	0.3391 (2)	0.0365 (7)	
C17	0.1145 (5)	0.5660 (2)	0.3820 (2)	0.0390 (7)	
H17	0.1940	0.5611	0.4518	0.047*	
C9	0.2888 (5)	0.0736 (3)	0.2913 (2)	0.0386 (7)	
C3	0.3849 (5)	0.3163 (3)	0.2945 (2)	0.0378 (7)	
C7	0.3920 (6)	−0.1113 (3)	0.3446 (2)	0.0453 (8)	
H7	0.3455	−0.1925	0.3474	0.054*	
C5	0.6977 (6)	0.0575 (3)	0.3885 (2)	0.0405 (7)	
H5	0.8608	0.0925	0.4207	0.049*	
C4	0.5884 (5)	0.2528 (3)	0.3452 (2)	0.0370 (7)	
C15	−0.1803 (6)	0.6487 (3)	0.2598 (2)	0.0431 (8)	
H15	−0.3077	0.6973	0.2454	0.052*	
C16	−0.0743 (5)	0.6358 (3)	0.3619 (2)	0.0401 (7)	
C11	0.3763 (5)	0.4240 (3)	0.3311 (2)	0.0385 (7)	
H11	0.5114	0.4544	0.3843	0.046*	
C12	0.1891 (5)	0.5030 (2)	0.3014 (2)	0.0378 (7)	
C8	0.2210 (6)	−0.0459 (3)	0.2943 (2)	0.0431 (7)	
H8	0.0587	−0.0818	0.2620	0.052*	
C13	0.0823 (6)	0.5165 (3)	0.1989 (3)	0.0442 (8)	
H13	0.1332	0.4755	0.1427	0.053*	
C14	−0.0998 (6)	0.5905 (3)	0.1791 (2)	0.0440 (8)	
H14	−0.1694	0.6010	0.1090	0.053*	
C2	0.2058 (5)	0.2476 (2)	0.2083 (2)	0.0376 (7)	
H2	0.0587	0.2908	0.1899	0.045*	
C21	0.3266 (5)	0.2321 (3)	0.1100 (2)	0.0387 (7)	
C22	0.2451 (7)	0.1373 (3)	0.0408 (2)	0.0517 (9)	
H22	0.1084	0.0819	0.0531	0.062*	
C6	0.6322 (6)	−0.0602 (3)	0.3915 (2)	0.0440 (8)	
H6	0.7491	−0.1062	0.4251	0.053*	
C26	0.5228 (6)	0.3134 (3)	0.0894 (2)	0.0493 (8)	
H26	0.5783	0.3797	0.1357	0.059*	
C23	0.3612 (8)	0.1222 (3)	−0.0467 (3)	0.0632 (11)	
H23	0.3042	0.0566	−0.0938	0.076*	
C24	0.5596 (7)	0.2024 (4)	−0.0654 (3)	0.0593 (10)	
H24	0.6417	0.1913	−0.1244	0.071*	
C25	0.6382 (6)	0.2991 (3)	0.0022 (3)	0.0558 (9)	
H25	0.7716	0.3556	−0.0114	0.067*	

### (E)-3-(3-Hydroxybenzylidene)-2-phenylchroman-4-one (II) Atomic displacement parameters (Å^2^)

**Table:**

	*U*^11^	*U*^22^	*U*^33^	*U*^12^	*U*^13^	*U*^23^
O1	0.0412 (11)	0.0325 (12)	0.0520 (13)	0.0005 (9)	0.0064 (10)	0.0075 (10)
O4	0.0394 (11)	0.0355 (12)	0.0525 (13)	0.0035 (10)	0.0017 (10)	−0.0001 (10)
O3	0.0553 (13)	0.0463 (14)	0.0510 (13)	0.0193 (11)	0.0022 (10)	0.0025 (11)
C10	0.0432 (16)	0.0317 (17)	0.0372 (15)	0.0082 (13)	0.0115 (13)	0.0044 (12)
C17	0.0413 (16)	0.0267 (16)	0.0469 (17)	0.0024 (13)	0.0014 (13)	0.0043 (13)
C9	0.0412 (16)	0.0344 (17)	0.0416 (16)	0.0037 (13)	0.0122 (13)	0.0036 (13)
C3	0.0397 (15)	0.0375 (17)	0.0372 (16)	0.0058 (13)	0.0083 (13)	0.0043 (13)
C7	0.0550 (19)	0.0309 (17)	0.0522 (19)	0.0020 (15)	0.0195 (15)	0.0020 (14)
C5	0.0438 (16)	0.0377 (18)	0.0419 (16)	0.0075 (14)	0.0108 (13)	0.0049 (13)
C4	0.0406 (16)	0.0363 (17)	0.0343 (15)	0.0048 (14)	0.0077 (13)	−0.0015 (12)
C15	0.0397 (16)	0.0345 (17)	0.0538 (18)	0.0054 (13)	0.0007 (14)	0.0081 (14)
C16	0.0428 (16)	0.0294 (16)	0.0478 (18)	0.0044 (13)	0.0063 (14)	0.0033 (13)
C11	0.0410 (16)	0.0328 (17)	0.0426 (16)	0.0055 (13)	0.0082 (13)	0.0057 (13)
C12	0.0383 (15)	0.0275 (16)	0.0469 (17)	0.0018 (13)	0.0053 (13)	0.0053 (13)
C8	0.0455 (16)	0.0343 (18)	0.0504 (18)	0.0023 (14)	0.0134 (14)	0.0009 (14)
C13	0.0481 (17)	0.0372 (18)	0.0479 (17)	0.0061 (14)	0.0079 (14)	0.0074 (14)
C14	0.0477 (17)	0.0373 (18)	0.0473 (18)	0.0074 (14)	0.0055 (14)	0.0087 (14)
C2	0.0374 (15)	0.0285 (16)	0.0463 (17)	0.0015 (12)	0.0063 (13)	0.0070 (13)
C21	0.0391 (15)	0.0376 (17)	0.0390 (16)	0.0077 (13)	0.0016 (13)	0.0051 (13)
C22	0.064 (2)	0.041 (2)	0.0476 (19)	0.0048 (17)	0.0052 (16)	−0.0032 (15)
C6	0.0511 (18)	0.0351 (18)	0.0492 (18)	0.0124 (14)	0.0117 (15)	0.0086 (14)
C26	0.0465 (18)	0.058 (2)	0.0433 (18)	0.0043 (16)	0.0078 (15)	0.0034 (16)
C23	0.087 (3)	0.057 (2)	0.046 (2)	0.023 (2)	0.0006 (19)	−0.0052 (17)
C24	0.059 (2)	0.079 (3)	0.0456 (19)	0.032 (2)	0.0082 (17)	0.0074 (19)
C25	0.0512 (19)	0.070 (3)	0.0469 (19)	0.0052 (18)	0.0094 (15)	0.0139 (18)

### (E)-3-(3-Hydroxybenzylidene)-2-phenylchroman-4-one (II) Geometric parameters (Å, º)

**Table:**

O1—C9	1.369 (3)	C15—H15	0.9500
O1—C2	1.453 (3)	C11—C12	1.475 (4)
O4—C4	1.234 (3)	C11—H11	0.9500
O3—C16	1.370 (4)	C12—C13	1.391 (4)
O3—H3	0.8400	C8—H8	0.9500
C10—C5	1.397 (4)	C13—C14	1.393 (4)
C10—C9	1.405 (4)	C13—H13	0.9500
C10—C4	1.460 (4)	C14—H14	0.9500
C17—C12	1.392 (4)	C2—C21	1.526 (4)
C17—C16	1.388 (4)	C2—H2	1.0000
C17—H17	0.9500	C21—C22	1.381 (4)
C9—C8	1.396 (4)	C21—C26	1.388 (4)
C3—C11	1.335 (4)	C22—C23	1.387 (5)
C3—C4	1.492 (4)	C22—H22	0.9500
C3—C2	1.499 (4)	C6—H6	0.9500
C7—C8	1.380 (4)	C26—C25	1.381 (4)
C7—C6	1.396 (4)	C26—H26	0.9500
C7—H7	0.9500	C23—C24	1.381 (5)
C5—C6	1.374 (4)	C23—H23	0.9500
C5—H5	0.9500	C24—C25	1.383 (5)
C15—C14	1.378 (4)	C24—H24	0.9500
C15—C16	1.383 (4)	C25—H25	0.9500
			
C9—O1—C2	115.9 (2)	C7—C8—H8	120.3
C16—O3—H3	109.5	C9—C8—H8	120.3
C5—C10—C9	118.8 (3)	C12—C13—C14	119.7 (3)
C5—C10—C4	121.1 (3)	C12—C13—H13	120.1
C9—C10—C4	119.8 (2)	C14—C13—H13	120.1
C12—C17—C16	120.9 (3)	C15—C14—C13	121.0 (3)
C12—C17—H17	119.5	C15—C14—H14	119.5
C16—C17—H17	119.5	C13—C14—H14	119.5
O1—C9—C8	117.0 (3)	O1—C2—C3	111.0 (2)
O1—C9—C10	122.8 (3)	O1—C2—C21	109.4 (2)
C8—C9—C10	120.2 (3)	C3—C2—C21	112.3 (2)
C11—C3—C4	118.3 (3)	O1—C2—H2	108.0
C11—C3—C2	127.3 (3)	C3—C2—H2	108.0
C4—C3—C2	114.4 (2)	C21—C2—H2	108.0
C8—C7—C6	121.1 (3)	C22—C21—C26	118.9 (3)
C8—C7—H7	119.5	C22—C21—C2	121.1 (3)
C6—C7—H7	119.5	C26—C21—C2	120.1 (3)
C6—C5—C10	121.2 (3)	C21—C22—C23	120.6 (3)
C6—C5—H5	119.4	C21—C22—H22	119.7
C10—C5—H5	119.4	C23—C22—H22	119.7
O4—C4—C10	123.2 (3)	C5—C6—C7	119.3 (3)
O4—C4—C3	121.2 (3)	C5—C6—H6	120.3
C10—C4—C3	115.6 (3)	C7—C6—H6	120.3
C14—C15—C16	119.6 (3)	C25—C26—C21	120.7 (3)
C14—C15—H15	120.2	C25—C26—H26	119.6
C16—C15—H15	120.2	C21—C26—H26	119.6
O3—C16—C15	118.4 (2)	C24—C23—C22	120.1 (3)
O3—C16—C17	121.8 (3)	C24—C23—H23	120.0
C15—C16—C17	119.8 (3)	C22—C23—H23	120.0
C3—C11—C12	129.9 (3)	C23—C24—C25	119.7 (3)
C3—C11—H11	115.1	C23—C24—H24	120.2
C12—C11—H11	115.1	C25—C24—H24	120.2
C17—C12—C13	118.9 (2)	C26—C25—C24	120.0 (4)
C17—C12—C11	117.1 (3)	C26—C25—H25	120.0
C13—C12—C11	124.1 (3)	C24—C25—H25	120.0
C7—C8—C9	119.4 (3)		

### (E)-3-(3-Hydroxybenzylidene)-2-phenylchroman-4-one (II) Hydrogen-bond geometry (Å, º)

**Table:**

*D*—H···*A*	*D*—H	H···*A*	*D*···*A*	*D*—H···*A*
O3—H3···O4^i^	0.84	1.89	2.728 (3)	172
C17—H17···O4^i^	0.95	2.49	3.184 (4)	130
C6—H6···O3^ii^	0.95	2.45	3.265 (4)	143
C11—H11···O4	0.95	2.43	2.807 (3)	103

Symmetry codes: (i) −*x*+1, −*y*+1, −*z*+1; (ii) *x*+1, *y*−1, *z*.
